# A Special View of What Was Almost Forgotten: p38δ MAPK

**DOI:** 10.3390/cancers13092077

**Published:** 2021-04-25

**Authors:** Débora Bublitz Anton, Rodrigo Gay Ducati, Luís Fernando Saraiva Macedo Timmers, Stefan Laufer, Márcia Inês Goettert

**Affiliations:** 1 Biotechnology Graduate Program, Universidade do Vale do Taquari (Univates), Lajeado, Rio Grande do Sul CEP 95914-014, Brazil; debora.anton@univates.br (D.B.A.); rodrigo.ducati@univates.br (R.G.D.); luis.timmers@univates.br (L.F.S.M.T.); 2 Medical Science Graduate Program, Universidade do Vale do Taquari (Univates), Lajeado, Rio Grande do Sul CEP 95914-014, Brazil; 3 Department of Pharmaceutical and Medicinal Chemistry, Institute of Pharmaceutical Sciences, Faculty of Sciences, University of Tuebingen, D-72076 Tuebingen, Germany

**Keywords:** MAPK13, p38δ MAPK, p38 delta, SAPK4, cancer, p38δ inhibitors

## Abstract

**Simple Summary:**

p38δ MAPK, as well as the other p38 isoforms, was proposed as a drug target for cancer therapy, owing to its critical roles in cellular signaling. However, herein, we show that p38δ inhibition may be therapeutically beneficial for treatment of specific cancer types, such as skin carcinoma, hepatocellular carcinoma (HCC), head and neck squamous cell carcinoma (HNSCC), cholangiocarcinoma (CC), and breast cancer. This review aims to discuss the roles of p38δ in cancer and summarize the findings of molecules with potential to inhibit p38δ in order to guide the search for new target cancer therapies based on p38δ inhibitors.

**Abstract:**

The p38δ mitogen-activated protein kinase is an important signal transduction enzyme. p38δ has recently emerged as a drug target due to its tissue-specific expression patterns and its critical roles in regulation of cellular processes related to cancer and inflammatory diseases, such as cell proliferation, cell migration, apoptosis, and inflammatory responses. However, potent and specific p38δ inhibitors have not been defined so far. Moreover, in cancer disease, p38δ appears to act as a tumor suppressor or tumor promoter according to cancer and cell type studied. In this review, we outline the current understanding of p38δ roles in each cancer type, to define whether it is possible to delineate new cancer therapies based on small-molecule p38δ inhibitors. We also highlight recent advances made in the design of molecules with potential to inhibit p38 isoforms and discuss structural approaches to guide the search for p38δ inhibitors.

## 1. Introduction

Mitogen-activated protein kinase (MAPK) cascades are signaling components that play a key role in transducing extracellular stimuli into cellular responses through phosphorylation of different substrates. MAPK signaling pathways control a variety of cellular processes, such as growth, differentiation, proliferation, survival, and death [1,2]. The mammalian family of MAPKs includes the extracellular signal-regulated kinase (ERK), c-Jun NH2-terminal kinase (JNK), and p38. Each of these cascades has at least three components: a MAPK kinase kinase (MAPKKK), a MAPK kinase (MAPKK), and a MAPK, which are phosphorylated and activated in sequence [3].

The p38 MAPK signaling pathways are activated by a wide variety of environmental and cellular stresses—for example, oxidative stress, hypoxia, and UV irradiation—or by inflammatory cytokines such as tumor necrosis factor (TNF)-α and interleukin (IL)-1β [4,5]. The upstream proteins MKK3 and MKK6 are responsible for activation of p38 MAPK [6]. MKK4 can also phosphorylate p38; however, this upstream protein is also an activator of JNK [7]. Once phosphorylated, p38 activates diverse cytoplasmic or nuclear protein substrates by catalyzing the transfer of the terminal γ-phosphate from the ATP to them. Substrates can be transcription factors, protein kinases, cytoskeletal proteins, and other cellular targets [5].

Four members comprise the p38 MAPK family: p38α (MAPK14), p38β (MAPK11), p38γ (MAPK12 or stress-activated protein kinases 3 - SAPK3), and p38δ (MAPK13 or SAPK4). Based on their amino acid sequence, different expression patterns, and sensitivity to chemical inhibitors, p38 isoforms can be separated into two subgroups: p38α and p38β represent one subgroup with 75% sequence identity, while p38γ and p38δ represent the other subgroup (62% and 61% sequence identity with p38α, respectively) [8]. While p38α is ubiquitously and abundantly expressed in cell lines and tissues, p38β is expressed at very low levels (compared to p38α), and its function seems to be redundant with p38α [9]. In contrast, p38γ and p38δ have a tissue-specific expression—for example, p38γ in skeletal muscle and p38δ in endocrine glands [4,5]. Each subgroup of p38 isoforms also has specific substrates: MAPK-activated protein kinase-2 and 3 (MK2 and MK3) are examples of substrates preferentially phosphorylated by p38α and p38β isoforms, while microtubule-associated protein Tau appears to be a better substrate for p38γ and p38δ [4,10,11]. Altogether, these observations suggest different cellular functions for each p38 isoform.

Due to the involvement of p38 MAPKs in cell transformation processes, such as unlimited proliferation, protection against apoptotic cell death, angiogenesis, tissue invasion, and metastasis, p38 isoforms emerged as drug targets for cancer therapy [2,12,13]. For a long time, p38α was the best-characterized isoform and the focus of numerous drug design projects. However, this has changed over time. p38α knockout mice die at the embryonic stage due to placental morphological defects, while p38β, p38γ, and p38δ knockout mice are viable and lack developmental defects [14,15,16,17]. Furthermore, mice lacking both p38γ and p38δ demonstrated no detrimental phenotypes and decreased innate inflammatory responses induced by lipopolysaccharide (LPS) [18]. Therefore, p38γ and p38δ isoforms may be more viable targets than p38α.

Although most scientific attention used to be focused on p38α, the p38δ isoform has recently emerged as a potential drug target due to its involvement in the regulation of cellular processes such as stress response, cytokines production, proliferation, migration, differentiation, and apoptosis [18,19,20]. In contrast to p38α, p38δ has distinct expression patterns depending on the tissue and, consequently, specific biological effects. Variable levels of p38δ expression were detected in endocrine tissues, including pituitary, prostate, salivary, and adrenal glands, and in pancreas, small intestine, testis, colon, kidney, and lung [10,21,22].

Physiological roles for p38δ have been reported in human disorders, such as neurodegenerative diseases [23], diabetes [17], inflammatory diseases [19,24,25], and cancer [26,27,28,29,30]. In cancer, p38δ appears to have contrasting roles: tumor suppressor or tumor promoter, which can change according to cancer and cell type studied [28,29,31,32,33]. Acting as tumor promoter, p38δ seems to also have a role in cancer invasion and metastasis [26,27]. Although these contrasting p38δ functions have been described, the specific roles played by p38δ on each cancer type have not been precisely defined.

This review aims to outline the current understanding of p38δ MAPK roles in cancer, for the purpose of determining the specific function of this enzyme in each cancer type and in tumor development. The restricted expression patterns and specialized functions of p38δ in cancer development, metabolic, and inflammatory diseases opens the possibility to delineate new target therapies based on small-molecule p38δ inhibitors. Although the design of p38 inhibitors is ongoing, p38δ specific inhibitors have not been discovered [34,35]. Therefore, this review also highlights recent efforts made to design molecules with potential to inhibit p38δ, summarizing findings and structural information on p38 family members to guide the search for specific p38δ inhibitors.

## 2. p38δ MAPK in Cancer-Suppressor or Promoter?

**Figure 1:**
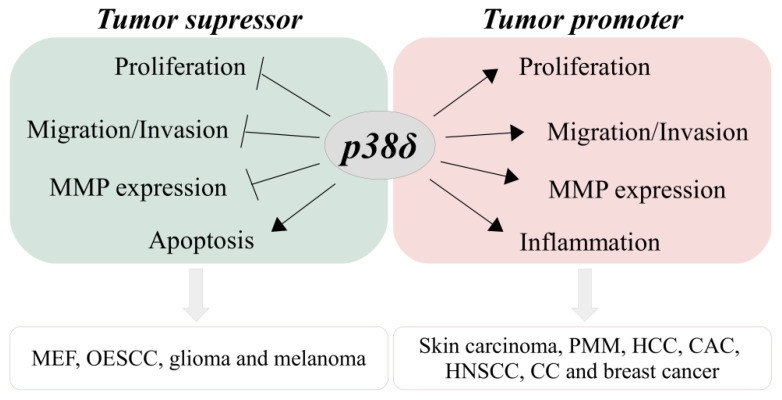
The contrasting roles of p38δ in cancer. MMP (matrix metalloproteinase), MEF (mouse embryonic fibroblasts), OESCC (oesophageal squamous cell carcinoma), PMM (pleural malignant mesothelioma), HCC, CAC (colitis-associated colorectal cancer), HNSCC, and CC.

The p38δ MAPK is activated in response to extracellular stimulus, such as cellular stresses and inflammatory cytokines, through dual phosphorylation on threonine (Thr180) and tyrosine (Tyr182) residues by the upstream kinases MKK6 or MKK3 [4,36]. However, it has been shown that MKK3 is the main activator of p38δ in fibroblasts cells that were exposed to cellular stresses, including hyperosmotic shock, ultraviolet radiation, and TNF- α cytokine, while MKK6 did not affect p38δ activation under these stimuli [6]. Activation of p38δ leads to phosphorylation of different substrates and, consequently, to several cellular responses. Some of these responses are associated with malignant transformation and cancer disorders, including cell proliferation, invasion, migration, and apoptosis [20,37]. However, p38δ appears to have both tumor suppressor and tumor promoter roles, depending on the cancer and cell type studied (Figure 1).

The first indication of p38δ as a tumor suppressor was observed in MEF. In these cells, p38δ appears to regulate processes that protect cells against malignant transformation, such as control of cell density, cell–cell contact inhibition, and cell migration [31]. p38δ^−/−^ MEFs, but not p38γ^−/−^ and wild-type (WT) MEFs, changed their morphology after reaching confluence and presented impaired contact inhibition. Moreover, either p38δ^−/−^ or p38γ^−/−^ MEFs proliferated faster than WT fibroblasts and displayed increased cell migration and matrix metalloproteinase 2 (MMP2) secretion [31]. Overexpression of MMPs in cells lacking p38δ was also observed in the human glioma-derived cell line SF767. In these cells, silencing of p38δ increased secretion of MMP2 and MMP9 and also promoted cell invasion [38]. MMPs are enzymes responsible for degrading several components of the extracellular matrix, and several studies associate their high expression levels with angiogenesis, cell motility, and metastatic spread of cancer [39,40]. Thus, the evidence that p38δ plays a role in the control of cell migration, invasion, and MMPs expression suggests that this enzyme is important for preventing cell transformation processes and decrease tumor progression.

The p38δ roles in control of cell proliferation and migration were also observed in oesophageal squamous cell carcinoma (OESCC). OESCC cell lines lacking p38δ expression proliferate faster than those endogenously expressing this isoform [32]. Nonetheless, reintroduction of p38δ into OESCC cells lacking p38δ expression resulted in the reduction of cell proliferation, migration, and number of colonies formed on soft agar, compared to WT cells [32]. Moreover, OESCC cells expressing p38δ are more sensitive to a combination chemotherapy than p38δ-deficient cells [41]. Therefore, in OESCC, p38δ appears to not only control proliferation but also regulates apoptosis.

A study revealed that epigenetic alterations may be responsible for suppression of p38δ expression in OESCC and, consequently, for tumor growth [42]. In OESCC cell lines, no mutations were identified in the MAPK13 gene sequence (encoding p38δ); however, hypermethylation of the MAPK13 promoter was found and associated with loss of p38δ expression [42]. Epigenetic alterations of the MAPK13 gene were also found in primary cutaneous melanoma, associated with tumor growth [43]. Approximately 67% of primary melanoma and 85% of metastatic melanoma presented promoter methylation of the MAPK13 gene, associated with downregulation of p38δ mRNA and protein expression [43]. Re-expression of p38δ in melanoma cells with MAPK13 promoter methylation reduced proliferation, indicating that p38δ may act as a tumor suppressor in melanoma cell lines [43]. Alternatively, the hypermethylation of MAPK13 gene promoter might, therefore, be an epigenetic cause of tumorigenesis promotion in cancer types where p38δ has antioncogenic roles.

In contrast to its tumor suppressor roles, p38δ also has tumor promoter roles, as evidenced in several types of cancers, such as skin carcinoma [29,44,45], pleural malignant mesothelioma (PMM) [46], HCC [47], colitis-associated colorectal cancer (CAC) [33], head and HNSCC [28,30], CC [26], and breast cancer [27]. The potential tumor promoter roles of p38δ are summarized in Figure 1.

Studies with the two-stage 7,12-dimethylbenz(a) anthracene/12- O-tetradecanoylphorbol-13-acetate (DMBA/TPA) chemical carcinogenesis model have evidenced a tumor promoter role for p38δ in skin squamous cell carcinoma (SCC). Specifically, p38δ-deficient mice were resistant to development DMBA/TPA-induced skin carcinoma, with a significant delay in tumor growth [44]. Moreover, both tumor number and size were significantly decreased in p38δ knockout mice compared to WT mice. This tumor reduction was associated with decreased levels of pro-proliferative ERK1/2-AP1 pathway and decreased activation of signal transducer and activator of transcription 3 (Stat3) [44]. Since Stat3 is an oncogenic transcription factor that plays a role in malignant conversion of skin tumors and is important for the phases of tumor initiation and promotion, p38δ might promote skin carcinogenesis (in part) through the activation of Stat3 [44,48]. The tumor promoter role of p38δ in DMBA/TPA induced skin tumorigenesis was confirmed by Zur and coworkers [45]. In mice, loss of p38γ, p38δ, or both reduced DMBA/TPA-induced skin tumor development [45].

Recently, mice lacking p38δ in epidermal keratinocytes or myeloid (immune) cells were employed to uncover the in vivo functional roles for p38δ in promotion of DMBA/TPA chemical skin carcinogenesis. Keratinocyte-specific p38δ ablation on mice did not influence the incidence or multiplicity of chemically induced skin tumors but reduced malignant progression in male and female mice in comparison to WT [29]. However, only in female mice, the tumors’ volume was increased during the TPA promotion stage, demonstrating a sex-specific mechanism of tumor development [29]. In contrast, in myeloid cells, intrinsic p38δ may be essential for the malignant progression of DMBA/TPA-induced skin tumors in males [29]. Myeloid cell-specific p38δ loss in male mice inhibited DMBA/TPA-induced skin tumorigenesis during both promotion and malignant progression stages, with reduced tumor incidence, multiplicity, and volume, compared to WT [29]. These evidences support that p38δ has tumor promoter roles in SCC and regulates skin carcinogenesis with different functions depending on the cellular context and stage of the tumor.

Additionally, p38δ has been reported as tumor promoter in pleural malignant mesothelioma (PMM), a rare and dangerous tumor mainly occurring in the pleural space of the lung. Three p38 MAPK isoforms are expressed by rat PMM cells, but only the p38δ isoform mediates proliferation stimulating signals [46]. In rat PMM cells stimulated with platelet-derived growth factor (PDGF)-BB, the induced proliferation was suppressed by downregulation of p38δ, evidencing the pro-proliferative role of p38δ [46]. The pro-oncogenic role of p38δ was also confirmed in gynecological cancer stem-like cells (CSCs)/cancer-initiating cells (CICs). CSCs/CICs are small subpopulations of tumor cells that are endowed with higher self-renewal, tumor-initiating, and differentiation abilities, becoming resistant to treatments [49,50]. CSCs/CICs isolated as aldehyde dehydrogenase (ALDH) high population, by RT-PCR, derived from HEC-1 cells (human endometrial carcinoma cells), MCAS and HTBoA cells (human ovarian cells), preferentially expressed the genes MAPK13 (p38δ), PTTG1IP, CAPN, and UBQLN2 over ALDH^low^ cells [51]. MAPK13 gene knockdown using siRNA reduced the ALDH^high^ population and abrogated tumor-initiating ability [51].

Another indication for a specific role of p38δ in promoting cancer progression has recently been demonstrated in HCC. This study identified a multikinase inhibitor, compound AD80, with p38γ and p38δ as its targets and antitumoral activity across a variety of HCC in vivo and in vitro models [47]. Moreover, growth inhibition assays and long-term clonogenic assays on HCC cell lines revealed that AD80 was more potent in these cells than Sorafenib, a compound commonly used in HCC therapy [47]. The potential pro-oncogenic role of p38γ and p38δ on aggressive HCC was confirmed by analysis of The Cancer Genome Atlas (TCGA) liver cancer dataset. Significantly better survival was correlated with low mRNA expression levels of p38γ and p38δ in HCC patient samples [47].

In some types of cancer, p38δ appears to promote carcinogenesis by regulating chronic inflammation, one of the hallmarks associated with tumor development and progression [18,20,33,52]. The tumor microenvironment is composed not only by cancer cells but also by tumor-promoting inflammatory cells that can release growth factors, chemokines, and cytokines responsible for tumor development [52]. Since formation of an inflammatory microenvironment is an essential component of all tumors, the expression profile of cytokines and chemokines can be indicative of tumor progression [53]. Some studies showed that both p38δ and p38γ modulate cytokine and chemokine production, immune cell migration, and T-cell activation [18,25,33,54,55]. For example, in LPS-stimulated macrophages from p38γ/δ-deficient mice, production of cytokines TNF-α, IL-1β, and IL-10 are severely reduced, whereas IL-12 and IFNβ production increases [18].

The increased release of inflammatory cytokines and chemokines by p38δ may enable formation of a proinflammatory microenvironment that sustains survival and growth of cancer cells. However, this inflammatory response is not exclusively activated by p38δ pathway but also relay on strong contribution of p38y. In CAC, a colon cancer subtype associated with inflammatory bowel disease, p38δ and p38γ promoted colon tumorigenesis through regulation of inflammatory responses [33]. When compared to WT controls, mice deficient in p38γ and p38δ exhibited reduced number of colon tumors, which correlated with a decrease in cytokines (IL-1β and TNF-α), chemokines production (MCP1, KC, and MIP-2), and in inflammatory cell infiltration in colon [33]. The same was observed in a TPA-induced skin tumor model, where p38γ/δ^−/−^ mice, in response to TPA, decrease the production of proinflammatory cytokines, including TNF-α, IL-1β, and IL-6, and decrease production of the chemokines KC and MIP-2, resulting in decreased neutrophil recruitment to skin [45]. p38δ was also associated with induction of IL-6 formation in human breast adenocarcinoma cell line MCF-7 [56].

Overexpression of the cytokines TNF-α, IL-1, and IL-6, which are enhanced by p38δ, has been associated with induction of angiogenesis, invasion, and metastasis of cancer cells [57,58,59]. Metastasis is a complex process that involves a series of steps. At some point in carcinogenesis, cells can lose cell–cell and cell-matrix contacts, detach from the primary tumor, and invade adjacent tissues. Through lymphatic or blood vessels, cells can spread to distant organs, where they can proliferate and create new colonies of cancer cells [52,60]. In addition to the tumor promoter role described in events of cell proliferation and tumor growth, p38δ has also been linked to activation of invasion and metastasis, another important hallmark of cancer [52]. p38δ seems to be responsible for loss of contact inhibition, cell invasion, cell migration, and increased metastatic lesions [26,27], which are important for detachment of primary tumor cells and, consequently, their growth in distant metastasis.

The first study attributing a role to p38δ in invasive migration of cancer cells was carried out on HNSCC. Inhibition of endogenous p38δ or p38α activity, the highly expressed p38 isoforms in HNSCC cell lines, effectively reduced invasion of HNSCC cells through collagen [30]. The inhibition of these isoforms also reduced mRNA expression of matrix metalloproteinases 1 and 13 (MPP1 and MPP13) [30]. Overexpression of MPP1 and MPP13 were correlated with increase of tumor aggressiveness and disease poor prognosis, due to their roles in the degradation of extracellular matrix, angiogenesis, and metastatic cascade [61,62,63,64]. Recently, all four p38 MAPK isoforms were found highly elevated in serum of HNSCC patients, compared to the control group; however, only p38α, p38β, and p38δ serum levels were decreased after therapy in follow-up patients [28].

The roles of p38δ in the promotion of cell migration and invasiveness were subsequently confirmed in CC [26]. p38δ expression was found to be upregulated in CC tissues when compared with cancer adjacent normal liver tissues and normal biliary tract tissues [26]. Knockdown of p38δ expression by siRNA transfection significantly inhibited motility of CC cells lines. Moreover, the knockdown of p38δ also led to a decrease in motility and invasiveness of CC cells, in a wound-healing assay and Matrigel invasion assay, respectively [26]. On the other hand, overexpression of p38δ increased invasiveness of CC cells lines, suggesting that p38δ might be related to CC metastasis [26].

p38δ has also been demonstrated to promote growth, migration, invasion, and metastasis of breast cancer by increasing cell proliferation and cell detachment. Comparison of p38δ protein expression in human breast cancer tissue with noncancer tissues revealed that, in normal tissues, p38δ was limited to ductal epithelium, while in breast cancer tissues, invasive ductal carcinoma sections had p38δ increased [27]. Likewise, in mouse breast cancer model MMTV-PyMT, p38δ was enhanced in breast tumor and lung tissue metastasis, compared to WT mice [27]. Loss of p38δ in MMTV-PyMT mice reduced tumor volume and, beyond 14 weeks, promoted a reduction in lung metastatic lesion exceeding 90% [27]. On the other hand, knockdown of p38δ in human breast cancer cell lines MCF-7 and MDA-MB-231 reduced cell proliferation in both cell lines and protected from detachment MDA-MB-231 cells stimulated with phorbol 12-myristate 13-acetate (PMA). Moreover, MDA-MB-231 cells lacking p38δ decreased cellular migration in a scratch-wound-healing assay [27]. These studies evidence that p38δ may not only regulate and affect growth of primary tumor but also promote the development of metastasis in breast cancer. Thus, p38δ is a promising target for treatment of early and advanced stages of breast cancer.

An association between activation of the p38δ pathway with tumor metastasis and growth was also observed in gastric cancer. The expression levels of gene G antigen 7B (GAGE7B), a member of the GAGE family that is involved in immune response, was found to be upregulated in poorly differentiated gastric cancer cell lines and in samples from advanced stage cancers (stages III-IV), compared to a well-differentiated cell line and samples from earlier stages (I-II) of gastric cancer, respectively [65]. Furthermore, GAGE7B was increased in metastatic tissues compared to nonmetastatic [65]. Gastric cancer cell lines overexpressing GAGE7B enhanced cell invasion, angiogenesis, and distant metastatic ability, and increased protein expression levels of p38δ, pMAPKAPK2, and pHSP27. In addition, the authors found that in gastric cancer samples with lymph node metastasis the miR-30c, a miRNA, was downregulated, while GAGE7B was upregulated. Overexpression of miR-30c in two gastric cancer cells suppressed both GAGE7B and p38δ expression, as well as inhibiting the migration and invasion ability of these cells, suggesting that the GAGE7B-induced p38δ activity might be related to gastric cancer metastasis [65]. Although some studies demonstrated that p38 MAPK signaling can activate HSP27 through MAPKAPK2 phosphorylation [66,67], there is no evidence that this occurs specifically by p38δ isoform. Therefore, the possibility that GAGE7B promotes tumor metastasis and growth of gastric cancer via activation of the p38δ/pMAPKAPK2/pHSP27 pathway requires further studies to be confirmed.

The majority of cancer-associated deaths are caused by metastatic disease rather than primary tumors [68]. Given the tumor promoter roles of p38δ, including cell proliferation, invasion, migration, and metastasis, this p38 isoform has emerged as a cancer drug target. The interest in targeted therapies with p38δ inhibitors is justifiable since the loss of this enzyme could not only prevent growth of primary tumor but also prevent the disease from progressing to an advanced stage with reduction of cell metastatic ability. Improving knowledge on this “forgotten” p38 isoform is an important advance toward treatment of some cancer types, especially for skin carcinoma, hepatocellular carcinoma, head and neck squamous cell carcinoma, cholangiocarcinoma, and breast cancer. Thus, for the types of cancers that p38δ has tumor promoting roles, it is possible to delineate new cancer therapies based on small-molecule p38δ inhibitors.

## 3. Searching Specific p38δ MAPK Inhibitors

Small-molecule inhibitors have become a valuable tool for the development of targeted therapies, since these compounds can be highly selective and effective against specific proteins. Several studies have been carried out with small-molecule inhibitors targeting protein kinases that control proliferation and death of cancer cells [34,69,70,71]. Therefore, these special molecules may potentially be more selective and less aggressive for cancer therapy than other compounds. At least 20 p38 inhibitor candidates are in clinical trials, some of which for the treatment of cancer [13,35]. Nonetheless, to date, there are no approved compounds with potential to inhibit p38 and several p38α inhibitors have failed in drug trials due to the high toxicity. Despite recent studies on isoform p38γ/δ-selective inhibitors for the treatment of cutaneous T-cell lymphoma (CTCL), none of the candidates in clinical trials focus specifically on the inhibition of the p38δ isoform (as reviewed by [35]).

The highly conserved ATP-binding site of the catalytic kinase domain is the major target of small-molecule kinase inhibitors. Like other protein kinases, p38 isoforms share a high degree of structural similarity within the ATP binding site, creating a challenge for designing selective inhibitors targeting this site [8,72]. Although p38 inhibitors differ on chemical structure and how they interact with the protein, most of them inhibit the enzyme by preventing ATP binding [72]. The challenge, therefore, relies on developing small-molecule inhibitors specific against p38δ, with minimal off-targets interactions, in order to decrease the chances of toxicity in later clinical trials. In order to explore less-conserved areas and improve inhibitor selectivity, it is necessary to know in detail the structure of p38 isoforms, particularly p38δ, as well as possible structural approaches that may guide this search.

As mentioned, all protein kinases share a similar structure; p38 isoforms, like other kinases, are folded into two subdomains: (i) an N-terminal subdomain (N-lobe) consisting of a five-stranded β-sheet and a long α-helix (αC-helix) and (ii) a large C-terminal subdomain (C-lobe) composed of six α-helices [8,73]. The cleft between the lobes forms the ATP-binding site. These two lobes are connected by a flexible hinge region that facilitates the movement between them, as well as being involved in ATP binding [73,74]. 

All p38 MAPKs exhibit two structural states: active and inactive, according to their phosphorylation state. The region that regulates p38 activity is the activation loop, where the conserved amino acids threonine (Thr) and tyrosine (Tyr) (TGY-motif) are localized [8,73]. Dual phosphorylation of the Thr and Tyr residues in p38 structures by an upstream kinase stabilizes the activation loop in an open conformation, increasing affinity for ATP and facilitating substrate binding [75,76]. Therefore, this dual phosphorylation locks the enzyme in an active conformation.

**Figure 2:**
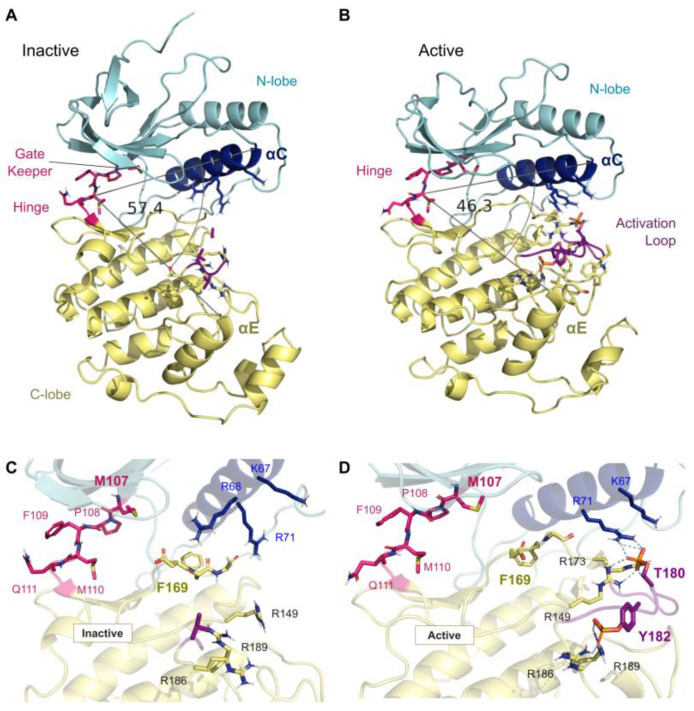
Overview of the p38δ in the (**A**) inactive (PDB ID 4YNO) and (**B**) active states (PDB ID 4MYG). Highlighting the N- and C-lobes in cyan and yellow, respectively, the hinge region in pink, activation loop in purple, and the alpha-C helix in dark blue. It is noteworthy that the active state has a tighter conformation with a smaller angle between the alpha-C and E helices. Highlights of the ATP-binding of both the (**C**) inactive and (**D**) active, with the double phosphorylation, states. Depicting the DFG motif into the inwards conformation (DFG-in) and inactive (DFG-out), respectively. The gate-keeper residue (Met107) is depicted as sticks. In the DFG-out state, Phe169 moves out of the pocket and expose a new site which type-II inhibitors can explore and bind. The Phe169 (F169) side chain is shown in both figures. Structure p38δ DFG-in (PDB ID 4EYM); structure p38δ DFG-out. Image generated in PyMOL.

The structure of p38δ, either in the active and inactive state, displays the standard structure of protein kinases, consisting of N- and C-lobes with a catalytic site between them [8] (Figure 2A,B). However, in the active form, the overall p38δ structure and catalytic site are more compact owing to interactions of phosphorylated Thr and Tyr with residues that coordinate the phosphorylation [8]. Although there is a high degree of sequence similarity between p38 isoforms, they rely on different residues to stabilize the negative charge introduced by the dual phosphorylation and to stabilize the active state (Figure 2C,D). Specifically, in p38δ, phosphorylation of residues Thr180 and Tyr182 is coordinated by R71, R173, and R186, which are similar in p38α and p38γ, as well as R149 and R189, which are only similar in p38γ [8]. Inhibitors that impair this coordination would likely stabilize the p38δ inactive conformation [8].

The design of specific p38 inhibitors could explore different structural elements of protein kinases to introduce potency and selectivity, since these enzymes are dynamic and can adopt multiple conformations [73]. An example is represented by the highly conserved motif Asp-Phe-Gly (DFG-motif) localized in the C-lobe of kinases that forms part of the ATP-binding site. According to the position of the DFG-motif, protein kinases can assume two major conformations: DFG-in and DFG-out, which can determine protein active or inactive state, respectively [73,77]. In the DFG-in conformation, protein kinases typically assume a structure where the Phe169 residue of the DFG-motif is packed into a hydrophobic pocket in the groove between the N- and C-lobes, forming a cleft where substrates can bind [76,78]. In addition, the Asp168 residue of the DFG-motif is at the beginning of the activation loop, where it can coordinate a magnesium ion bind that interacts with an oxygen atom of the β-phosphate of ATP [73,76]. A DFG-in conformation was already observed in the p38δ structure. When p38δ is activated by dual phosphorylation, the side chain of Asp168 rotates, which prevents the movement of Phe169 side chain out of the hydrophobic pocket [8] (Figure 2A).

On the other hand, in the DFG-out conformation, Asp168 flips and Phe169 moves out of the DFG-pocket [77]. This DFG-flip induces structural changes in the activation loop of the enzyme, obstructing part of the ATP binding site and exposing an additional hydrophobic site in the kinase [47,79]. The position of DFG-motif in DFG-out is incompatible with ATP binding; therefore, the protein is at the inactive state [79]. Furthermore, in DFG-out (Figure 2C), the unoccupied DFG-pocket becomes more accessible for inhibitor binding.

p38 MAPK inhibitors were designed following the DFG-out and DFG-in binding mode. According to their mechanisms of inhibition, p38 inhibitors can be classified into three main categories: type-I, type-II, and type-1½ [34,35]. Type-I inhibitors are ATP-competitive compounds that occupy only the adenine-binding pocket, forming from one to three hydrogen bonds with the backbone residues of the hinge region [78,80]. These ligands typically bind in a DFG-in conformation, recognizing the active state. The compounds SB203580 and Skepinone-L are good examples of highly potent inhibitors that bind to p38α in DFG-in mode [81,82]. In contrast, inhibitors defined as type-II bind in the DFG-out state and occupy, in addition to the ATP-binding site, the new hydrophobic site accessible in the inactive form due to the rotation of DFG-motif [78]. Type-II inhibitors bind to the ATP site through hydrogen bonds with the hinge region and also exploit hydrogen bonding and hydrophobic interactions within the hydrophobic site, stabilizing the inactive state [83]. The compound BIRB796 is an example of type-II inhibitor of p38 that binds in the DFG-out mode of the enzyme and indirectly competes with ATP [79].

The type-1½ is a subtype of type-I, since inhibitors of this class bind to the ATP-binding site like type-I compounds. However, these ligands also extend into a mainly hydrophobic back cavity of the ATP site where they make specific interactions similar to type-II inhibitors, typical of the DFG-out state [78]. Therefore, type-1½ inhibitors are capable of binding the kinase in the activated and inactivated states. The back cavity that type-1½ ligands explore is not occupied by the ATP co-substrate, and its size depends on the gatekeeper residue, an important residue of kinases which is located in the first position of the hinge region [78]. Kinases with gatekeeper residues with bulky side chains (e.g., Met, Phe, or Leu) have a small back cavity that reduces the space that inhibitors could explore, while small gatekeeper residues (e.g., Thr or Val) increase the size of the cavity [78]. An improvement in selectivity and potency of p38α MAPK inhibitors was observed when they explored this new binding mode [84,85]. Nonetheless, to date, there has been no type-1½ inhibitor described for p38δ.

**Figure 3:**
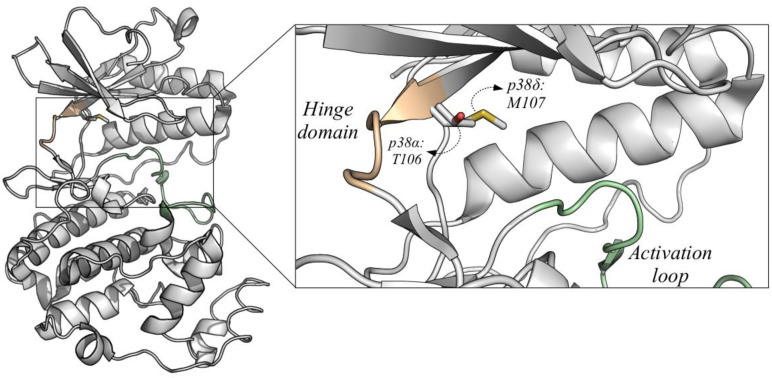
Structure of p38δ and the gatekeeper residue. Ribbon diagram of p38δ (PDB 4myg) showing the hinge domain in orange and the activation loop in green, regions near the ATP binding pocket. The p38α gatekeeper residue (threonine, T106) is shown in the structure of p38δ to compare with the p38δ gatekeeper residue (methionine, M107). Methionine is a bulky residue and takes up more space, which might impair the binding and selectivity of inhibitors. Therefore, it is important to consider this difference for design of p38δ inhibitors. Image generated in PyMOL.

The potency and selectivity of p38 inhibitors may be better in the p38 isoforms with smaller gatekeeper residues. Instead of threonine (Thr) present in p38α and p38β, p38δ and p38γ possess as gatekeeper residue a bulky methionine (Met), which might interfere in the binding of some types of compounds (Figure 3). For example, p38α and p38β isoforms are susceptible to inhibition by pyridinyl imidazoles, such as compounds SB203580 and SB202190, while p38δ and p38γ are insensitive [10,86,87]. A single amino acid substitution in the gatekeeper residue in p38δ and p38γ, replacing Met by Thr, makes them sensitive to SB203580 [87]. Kondoh and coworkers described an approach to guide a primary screening for selective inhibitors against p38δ and p38γ based on this gatekeeper residue variation [86]. The approach consists of a comparative screening using chemical arrays aiming to identify specific inhibitors that specifically bind to p38α-T106M mutant, which mimics the p38δ/p38γ gate-keeper configuration, and not to p38α wild-type [86]. The chemical arrays used in the study contained thousands of compounds and two fluorescent markers, and these arrays were treated with GST-p38α wild-type or GST-p38α^T106M^ mutant. Since the probe had two markers, specific binders of GST-p38α^T106M^ were detected as red spots, while specific binders of GST-p38α were green [86]. Therefore, it was possible to detect compounds that bind specifically to p38δ and p38γ, as these proteins naturally have a Met as the gatekeeper residue.

The chemical array described to screening selective p38δ and p38γ inhibitors identified the compound SU-002 as an inhibitor of p38α^T106M^ mutant, and in vitro kinases assays confirmed the potential of SU-002 to preferentially inhibit p38 isoforms with gatekeeper residue Met over Thr [86]. SU-002 is a compound with a 2-(7-chloroquinolin-4-yl) sulfanyl-1,3,4-thiadiazole (CQT) structure that has attachment of a long alkyl chain (octanamide) (Table 1). To improve this favorable structure to inhibit p38δ and p38γ, the compound SU-005 was synthesized changing the octanamide of SU-002 by an oct-2-enamide attached to CQT structure (Table 1) [86]. SU-005 specifically decreased p38δ and p38γ activity both in vitro and in cells lines and did not inhibit p38δ with a Met107Thr mutant, suggesting that this compound may be a specific inhibitor of p38δ and p38γ owing to the presence of the gatekeeper residue Met [86]. The authors also suggested that an inhibitor structure with an octanamide or oct-2-enamide attached to the CQT structure is responsible for targeting the Met gatekeeper residue and, consequently, preferentially inhibits p38δ and p38γ.

The gatekeeper methionine of p38δ may also be responsible for its poor sensitivity to the type-II inhibitor BIRB796, since this compound has a naphthalene group that sterically clashes with this large residue in p38δ structure [19]. Aiming to obtain better p38δ inhibitors, three compounds—61, 62, and 124—analogs of BIRB796 have been designed, replacing its naphthalene group to a smaller monoaryl ring (Table 1) [19]. Two of them, compounds 61 and 62, presented the expected binding in the DFG-out state of p38δ and highly inhibited p38δ activity. On the other hand, compound 124 adopted a DFG-in binding mode and showed lower inhibitory activity against p38δ. However, all the three designed compounds also inhibited the activity of p38α, which shows that the changes made in their structures were not sufficient to interfere with the binding to other p38 isoforms [19]. Although the compounds were not specific to p38δ, they are a step forward in the knowledge about what type of inhibitors will inhibit with higher potency to p38δ. While BIRB796 had an IC_50_ of 1968 nM against p38δ, compounds 61 and 62 presented IC_50_ values of 620 nM and 281 nM, respectively [19]. Thus, it appears that potent p38δ inhibitors can be designed following the DFG-out binding mode, with structures that do not clash with the Met gatekeeper residue in the hinge region of p38δ.

Yurtsever and coworkers also observed that compounds with binding in the DFG-out conformation of p38δ had higher potency than those with binding in the DFG-in conformation [88]. The authors characterized two new p38δ inhibitors, compound 58 and 117, and compared them to compounds previously described, 61 and 124 (Table 1). Similar to compound 61, compound 58 showed a DFG-out binding mode and IC_50_ in the nanomolar range. In contrast, compound 117 displayed a DFG-in binding mode (similar to compound 124) and IC_50_ in the micromolar range [88]. Moreover, the DFG-out inhibitors (compounds 58 and 61) displayed slower dissociation rates as compared to DFG-in inhibitors (compounds 117 and 124), which may lead to improvement in inhibitor activities [88].

A screening for type-II kinase inhibitors in a panel of five models of HCC cell lines was performed to find potential treatments for hepatocarcinoma. The study identified AD80 as a highly potent compound in HCC cell lines compared to Sorafenib based on growth inhibition and clonogenic assays [47]. AD80 also proved to be more potent than Sorafenib in a tumor xenograft model, with higher animal survival and increase in reduction of tumor growth rate per day [47]. Proteomic screening and biochemical analysis revealed that p38δ and p38γ were the direct targets of AD80 [47]. Interestingly, the gatekeeper residue appears to control AD80 selectivity to both isoforms of p38, and it seems to prefer enzymes with larger gatekeeper residues. Point mutations in p38α and p38β replacing Thr106 for Met were performed, as well as substitution of Met107 to Thr in p38δ (p38δ^M107T^) and Met109 to Thr in p38γ (p38γ^M109T^). Biochemical melting temperature assays revealed that AD80 binds more favorably in p38α^T106M^ and p38β^T106M^ mutants than in p38δ^M107T^ and p38γ^M109T^ mutants [47].

A recent study developed a peptide inhibitor against p38δ with the proposal to be used in the treatment of HNSCC, named WFYH (Table 1) [28]. Virtual screening was performed with a tetrapeptide library and p38δ as the target protein, and ten tetrapeptides were selected for postdocking analysis. The peptide WFYH showed the best docking scores and good binding affinity for p38δ in contrast to the other p38 isoforms [28]. WFYH at 75 µM inhibited 77% of p38δ activity, based on ELISA assays using activating transcription factor 2 (ATF-2) as a substrate [28]. The authors suggested that this peptide may have greater binding affinity for p38δ over the other p38 isoforms owing to the strong noncovalent π–π interaction that the benzene ring of Trp of WFYH made with Phe169 residue (in DFG-motif) of p38δ and that this interaction also prevents Phe169 from moving.

An approach involving targeting protein kinases upstream of p38δ MAPK could be an alternative strategy to achieve specific inhibition of p38δ [89]. The upstreamMKK3 is known to be a specific activator of all p38 MAPK isoforms. Nonetheless, in colorectal cancer (CRC) cell lines, MKK3 has been identified as a specific activator of the p38δ isoform [90]. MKK3 was found highly expressed in the late stage of CRC, and its overexpression was correlated to poor prognosis [90]. Moreover, in CRC lines, MKK3 silencing increased the efficacy of the chemotherapeutic 5-FU, an effect that was associated with inhibition of p38δ [90]. It was also observed that when CRC lines were exposed to 5-FU, p38δ phosphorylation and activation were induced by MKK3; therefore, this molecular signaling may be responsible for decreasing the efficacy of the molecule [90]. p38δ knockdown in CRC lines also demonstrated anti-proliferative effects; however, as presently, p38δ specific inhibitors do not exist, it has been proposed that MKK3 inhibitors may be a potential treatment for colorectal cancer owing to the inhibition of MKK3/p38δ prosurvival signaling [89,90]. The benefit of MKK3 inhibitors over p38 inhibitors lacking specificity can be based on the fact that, in CRC, while p38δ has tumor promoter roles, p38α seems to be a tumor suppressor [89]. Thereby, the selectivity of p38 inhibitors is crucial for cancer therapeutic purposes.

## 4. Conclusions

p38δ MAPK isoform for a long time was practically forgotten or did not arouse the interest of researchers, mostly because it is not an abundant protein expressed in all tissues as p38α. However, as demonstrated in this review, p38δ has some advantages as a drug target due to its restricted expression pattern. Furthermore, p38δ has several important biological roles, especially in cancer disease where it seems to be a tumor suppressor or promoter, depending on the cancer type. There are several evidences showing that p38δ is responsible for proliferation, migration, and invasion of tumor cells, which is critical for tumor progression and formation of metastasis. Therefore, p38δ inhibition is a potential therapeutic strategy for the treatment of cancer, not only for those in the early stage, but also for advanced stages of the disease. In this context, it is important to take into account that the contrasting roles of p38δ could bring some prejudicial responses when this isoform is inhibited, since it can stop tumor progression in a tissue, while leading to a malignant transformation in others. The selectivity of p38δ inhibitors is important and require special attention, as in some types of cancer the isoforms of p38 (among themselves) may have contrasting roles.

Although studies have demonstrated that targeting DFG-out binding mode may have some advantages in p38 inhibition, it has also been shown that p38 inhibitors can bind equally well to both DFG-in and DFG-out mode, with differences in selectivity and potency. Specifically, for p38δ, it looks like inhibitors with a DFG-out binding mode are more potent than those with a DFG-in binding mode. This evidence leads to the conclusion that type-II inhibitors, or maybe type-1½, may be favorable in inhibiting p38δ since they could bind in a DFG-out state. Moreover, the structure of compounds with the proposal to inhibit p38δ needs to consider that this isoform has a bulky methionine as gatekeeper residue, which might impair the binding and selectivity of the inhibitor. Despite the limited success to obtain inhibitors specific to p38δ, this p38 isoform remains a potential candidate for the development of new targeted therapies for cancer. The efforts to design a potent and selective inhibitor of p38δ are important, since the lack of these compounds limits knowledge on p38δ biological functions, as well as delaying the development of new targeted therapies based on p38δ inhibition.

## Figures and Tables

**Table 1:** Structures of potential p38δ inhibitors.

**Name**	**Structure**	**Binding Mode ^1^**	**Reference**
SU-002	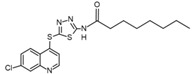	-	[86]
SU-005	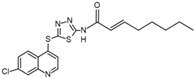	-	[86]
61	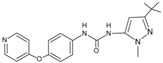	DFG-out	[19]
124	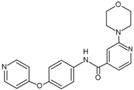	DFG-in	[19]
58	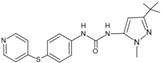	DFG-out	[88]
117	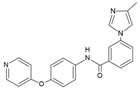	DFG-in	[88]
AD80	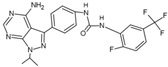	DFG-out	[47]
WFYH	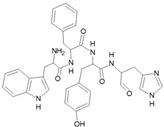	-	[28]

^1^ Compounds without binding mode is because it was not specified in the study.
